# Generation of skeletal muscle cells from pluripotent stem cells: advances and challenges

**DOI:** 10.3389/fcell.2015.00029

**Published:** 2015-05-13

**Authors:** Ramzey Abujarour, Bahram Valamehr

**Affiliations:** Fate Therapeutics Inc.San Diego, CA, USA

**Keywords:** skeletal muscle cells, satellite cells, skeletal muscle, pluripotent, IPSC, pax7, MyoD, myogenic differentiation

## Abstract

Human pluripotent stem cells (hPSCs) possess unlimited proliferative potential while maintaining the ability to differentiate into any cell type including skeletal muscle cells (SMCs). hPSCs are amenable to genetic editing and can be derived from patient somatic cells, and thus represent a promising option for cell therapies for the treatment of degenerative diseases such as muscular dystrophies. There are unresolved challenges however associated with the derivation and scale-up of hPSCs and generation of differentiated cells in large quantity and high purity. Reported myogenic differentiation protocols are long, require cell sorting and/or rely on ectopic expression of myogenic master regulators. More recent advances have been made with the application of small molecules to enhance the myogenic differentiation efficiency and the identification of more selective markers for the enrichment of myogenic progenitors with enhanced regenerative potential. Here we review the field of myogenic differentiation and highlight areas requiring further research.

## Introduction

Skeletal muscle has a great capacity to regenerate following muscle wasting caused by trauma or disease. This regenerative potential is attributed primarily to skeletal-muscle resident stem cells called satellite cells (SCs) that are distinguished by their expression of PAX7 and their location between the basal lamina and the sarcolemma of myofibers (Wang and Rudnicki, 2012). In Duchenne muscular dystrophy (DMD), a progressive muscle wasting caused by an absence of functional Dystrophin protein, SCs are exhausted following many rounds of muscle degeneration and regeneration (Heslop et al., 2000). Hence, SCs and their progeny (myoblasts) have been considered as a promising candidate for cell replacement therapy for DMD and other types of muscle disease (Skuk et al., 2014). There are several major limitations to the use of SCs as a cell therapy including their paucity, difficulty to isolate, limited proliferative capacity and loss of *in vivo* regenerative potential upon *ex vivo* expansion (Montarras et al., 2005). In addition, genetic engineering of SCs is challenging without immortalization (Ousterout et al., 2013) or sustained association with muscle fibers in *ex vivo* culture (Marg et al., 2014).

hPSCs such as human embryonic stem cells (hESCs) and induced pluripotent stem cells (hiPSCs) possess two unique features that distinguish hPSCs from adult stem cells: unlimited proliferative potential and ability to differentiate into any cell type including SMCs. With the added benefits of being amenable to genetic editing (Li et al., 2015), hPSCs hold great promise as a source of cell replacement therapy for degenerative diseases like DMD. hiPSCs are generated by the over-expression of a combination of master pluripotency regulators (e.g., *OCT4 (POU5F1), SOX2, KLF4, MYC, LIN28*, and *NANOG*), and can be generated from a variety of somatic cell types from healthy donors and patients (Takahashi et al., 2007; Yu et al., 2007; Abujarour et al., 2014). hiPSCs thus evade the ethical and technical hurdles that limit the clinical use of hESCs. Recent advances have been made in the generation, defined culture and scale up of hiPSCs which are prerequisite steps needed for any translation into the clinic. For example, several non-integrating methods have been developed to generate more clinically-compatible transgene-free hiPSCs, albeit at reduced efficiencies (Wu et al., 2013). To enhance reprogramming efficiency, a combination of small molecules and more stringent cell sorting strategies were shown to enable transgene-free single cell generation and maintenance of hiPSCs under feeder cell-free conditions (Valamehr et al., 2012, 2014; Abujarour et al., 2013). However, major challenges associated with the clinical use of hPSCs remain, including the ability to efficiently generate functional and engraftable differentiated cells, including SMCs, in large quantity and high purity. Here we will review the progress toward the differentiation of hPSCs into SMCs.

## Generation and selection of myogenic cells from hPSCs

In an early attempt to generate SMCs, hESCs were co-cultured with stromal cells (OP9) to induce their differentiation into multipotent mesenchymal precursors (Barberi et al., 2005). The hESCs-derived mesenchymal precursors were enriched based on their expression of CD73 and differentiated further into SMCs that formed multinucleated myotubes when co-cultured with mouse myoblastic line C2C12. Co-culture with stromal cells was shown later by the same group to be unnecessary for generation of SMCs from hESCs as prolonged culture of hESCs in fetal bovine serum (FBS) and more elaborate enrichment stragety could compensate for lower differentiation efficiency (Barberi et al., 2007). With selection of mesenchymal precursors by virtue of CD73 expression followed by expansion and another round of cell sorting using the more skeletal muscle-specific marker CD56 (NCAM), it was possible to obtain a culture enriched with MYOD+ cells. Without the need to co-culture with C2C12, serum derprivation by itself efficiently induced CD56+ cells to express the mature myogenic marker MYOGENIN and undergo cell fusion to form multi-nucleated myotubes capable of spontenous twitchning. In addition of the MYOGENIN+ mature SMC fraction, a small fraction of myogenic precurosor cells expressing PAX7 was also detected. To test engraftment potenital *in vivo*, luciferase-expressing hESC-derived CD73+/CD56+ cells were transplanted into cardiotoxin-injured tibialis anterior (TA) muscles of immunocompromised (SCID/Beige) mice and monitored noninvasively. A substantial fraction of cells did not survive the first week of transplantation due to cell death. However, the transplanted cells stablized and persisted over a period of 6 months forming 7% of the myofibers in the host muscle as determined by human-specific laminin. The long-term engraftment of the hESC-derived SMCs suggests the persistence of myogenic progenitor cells that can contribute to prolonged rounds of regneration. In a different approach, myogenic differentiation of hESCs was induced by formation of floating cell spheres called embryoid bodies (EBs) followed by attachment on gelatin in media containing FBS and epidermal growth factor (EGF) (Zheng et al., 2006). After 4 weeks in culture, expression of early myogenic markers MET (HGFR), PAX7 and MYOD was detected but cells failed to express mature myogenic markers or form myotubes, highlighting the poor efficiency of spontaneous myogenic differentiation methods. Nonetheless, transplantation of these early myogenic cells into cardiotoxin-injured TA muscles of immunocompromised (NOD-SCID) mice allowed further maturation and incorporation of some transplanted cells into the host myofibers, indicating that the *ex vivo* differentiation conditions were missing critical myogenic induction cues. A similar EB- and serum-based approach was optimized further through elaborate dissociation and re-cultivation of EBs leading to better myogenic differentiation of hESCs and hiPSCs *in vitro* and *in vivo* (Awaya et al., 2012). Significantly, teratoma formation was not observed in any of the grafted mice in the studies mentioned above.

## Transgenic methods for myogenic differentiation of hPSCs

Reported methods for the generation of SMCs from hPSCs are invariably inefficient, overlong and usually involve cell sorting for enrichment, especially if the small fraction of myogenic progenitors/SCs with the highest regenerative potential is desired. To overcome these limitations, several studies described the use of ectopic expression of myogenic specification factors in hPSCs or hPSC derivatives as a means to enhance and accelerate the myogenic differentiation process. One such myogenic factor is MYOD which is one of the earliest examples of “master” transcription factors that are capable of transdifferentiating cells from one lineage (e.g., fibroblasts) into another (skeletal muscle) (Davis et al., 1987). Benefitting from this approach, Tedesco and colleagues demonstrated the potential to generate engraftable myogenic cells from genetically-corrected hiPSCs derived from patients with limb-girdle muscular dystrophy 2D (LGMD2D), a disease caused by mutations in the gene encoding α-sarcoglycan (Tedesco et al., 2012). The patient-specific hiPSCs were differentiated into mesodermal progenitors (mesoangioblast-like or HIDEMs) that were genetically corrected with a lentiviral vector carrying the gene encoding human α-sarcoglycan under the control of a muscle-specific promoter. To induce terminal differentiation, HIDEMs were transduced with a lentiviral vector containing tamoxifen-inducible MYOD (MYOD-ER). Following transplantation of the corrected MYOD-ER–expressing HIDEMs in the TA muscles of immune-deficient Sgca-null/SCID/beige mouse and daily administration of tamoxifen (for 7 days), substantial engraftment and formation of donor-derived muscle fibers expressing α-sarcoglycan were observed. In addition, intra-arterial transplantation of the genetically corrected HIDEMs led to colonization of the skeletal muscle downstream of the injection site. Another muscular dystrophy, Miyoshi myopathy, was also recently modeled using ectopic expression of MYOD in patient-derived hiPSCs followed by phenotypic rescue by expression of full-length DYSFERLIN which is mutated in the disease (Tanaka et al., 2013).

In a related work, Goudenege and colleagues attempted initially the direct myogenic conversion of hESCs into SMCs without passing through a mesodermal stage through the ectopic expression of MYOD using adenoviruses but the efficiency of myogenic conversion was unexpectedly very poor (Goudenege et al., 2012). By differentiating hESCs first to mesenchymal-like CD73+ cells followed by MYOD transduction, the authors achieved a much higher myogenic conversion. Upon transplantation into cardiotoxin-injured TA muscles of immunocompromised mouse model of DMD (Rag/mdx), a high level of engraftment in host muscles was observed. Interestingly, the total number of human myofibers was significantly higher in the muscles injected with the hPSC-derived SMCs than in muscles injected with the same number of control human myoblasts. This is a surprising finding as MYOD is a marker of activated SCs (i.e., myoblasts) and suggests that the MYOD-expressing hPSC-derived myoblasts have a higher engraftment potential than their primary counterparts. Again, no teratoma was observed in the muscles transplanted with hPSCs or with hPSC-derived cells (Goudenege et al., 2012). A separate study attributed the observed resistance of hPSCs to MYOD-mediated induction of myogenesis to the absence of the chromatin-modifying factor BAF60C. When hESCs were transduced with exogenous BAF60C followed by MYOD, efficient conversion of hESCs into myosin heavy chain (MYHC)-positive SMCs was observed (Albini et al., 2013). Alternatively, we have shown that direct myogenic conversion of ground-state hiPSCs from healthy or DMD donors into SMCs can be achieved within 3–5 days of Doxycycline-induced expression of MYOD (Abujarour et al., 2014). It is unclear why MYOD-directed myogenesis in our system did not require ectopic expression of BAF60C. One possibility is that we cultured hiPSCs in a small molecule-supplemented media under feeder cell-free conditions that maintained hiPSCs in a ground state resembling the naïve stage of pluripotency (Valamehr et al., 2014) and possibly enhanced a more efficient MYOD-mediated myogenic conversion. A direct MYOD-mediated differentiation method has also been successfully demonstrated using MYOD-encoding modified RNA and hiPSCs derived using similar mRNAs encoding reprogramming factors, indicating that iPSCs and specialized derivatives could be generated using safer non-integrating methods (Warren et al., 2010).

The ectopic expression of MYOD is an attractive approach as it dramatically accelerates the generation of SMCs from hPSCs (from > 5 weeks to few days) and might have a safer profile due to its tumor suppression activity (Dey et al., 2013). However, the MYOD-based method yields mostly mature SMCs that are predicted to have limited proliferative and regenerative capacity compared with immature/inactivated SCs (Montarras et al., 2005). However, with the ability of MYOD-induced hPSC-derived myoblasts to engraft efficiently into injured muscles of immunocompromised mice (Goudenege et al., 2012), a direct comparison of the engraftment potential of hPSC-derived SMCs generated using the MYOD method vs. other methods will be helpful.

Using a different approach and learning from their previous studies in mouse (Darabi et al., 2011a,b), Darabi and colleagues relied on the ectopic expression of the myogenic factor PAX7, archetypical marker of adult SCs, to generate myogenic progenitors/SCs from hPSCs, (Darabi et al., 2012; Wang and Rudnicki, 2012). hiPSCs and hESCs were modified with a doxycycline-inducible lentiviral vector encoding PAX7 and GFP (separated by IRES). Unlike the direct MYOD-mediated approach, hiPSC/hESCs were first differentiated into a paraxial mesoderm stage (marked by high *BRACHYURY* expression) using EB based method and serum-rich media, followed by PAX7 (and GFP) induction and cell sorting based on GFP expression. The sorted cells showed progenitor-like characteristics exhibiting high proliferative capacity and minimal signs of terminal differentiation when cultured in the presence of Dox, FGF, and serum. Upon culture in differentiation media (with 2% horse serum) and withdrawal of Dox and FGF, the myogenic progenitors differentiated efficiently into multinucleated myotubes with only rare cells maintaining PAX7 expression. To test their myogenic potential *in vivo*, the progenitor cells expressing transgenic PAX7 were injected into cardiotoxin-injured TA muscles of immunocompromised DMD mouse model (NSG-mdx4Cv). The hPSC-derived progenitors led to considerable engraftment that was much higher than that observed with control human primary myoblasts. Injection with the hPSC-derived progenitors led to restoration of Dystrophin expression in the dystrophic muscles and significant functional improvement as demonstrated by increased isometric tetanic force, absolute force, and specific force. Interestingly, transplantation of primary myoblasts did not result in improvement of any of these functional parameters. In addition, PAX7+ hPSC-derived progenitor cells were detected within the sarcolemma of myofibers, indicating that hPSC-derived progenitors seeded the SC compartment. This contribution to the stem cell niche was associated with long-term engraftment of hPSC-derived progenitors that was sustained close to a year after transplantation into NSG mice.

## Use of small molecules

Non-transgenic methods for the differentiation of hPSCs, whether as a monolayer culture or as EBs, into SMCs remain inefficient and overlong. One promising approach to improve the myogenic differentiation efficiency of hPSCs is the use of small molecules to inhibit or activate relevant signaling pathways. Similar chemical approaches have been applied successfully to enhance the efficiency of hiPSC generation, culture (Valamehr et al., 2012, 2014) and differentiation into multiple lineages (Xu et al., 2013b). Two groups recently reported enhanced myogenic differentiation of hESCs/hiPSCs through the sequential treatment with a WNT activator, the small-molecule glycogen synthase kinase-3 (GSK-3) inhibitor CHIR99021, and fibroblast growth factor 2 (FGF2) in serum-free media (Borchin et al., 2013; Shelton et al., 2014). Interestingly, the two methods reported relatively high levels of PAX7+ myogenic progenitor cells ranging between 18% by week 5 (Borchin et al., 2013) and 43% by week 7 (Shelton et al., 2014). Combined with their two-step differentiation method, Borchin and colleagues described a stringent cell sorting strategy for the enrichment of PAX3+/PAX7+ myogenic progenitors based on the negative selection markers HNK (neural crest) and nicotinic acetylcholine receptor (AChR; mature skeletal marker) and positive selection markers CXCR4 and MET (Borchin et al., 2013). The new cell sorting strategy led to efficient enrichment of PAX3+/PAX7+ cells, with MET appearing to be the more critical marker in this sorting strategy. This is the first example demonstrating the ability to obtain skeletal myogenic PAX7+ progenitor cells in high purity from hPSCs without genetic modification. It will be interesting in the future to explore how the engraftment potential of these purified myogenic progenitors compare to that of primary myoblasts isolated and *ex vivo* expanded from human muscle.

In order to identify novel chemical modulators of the skeletal muscle lineage, Xu and colleagues carried out a high-throughput image-based screen using blastomere cells from a myf5-GFP and mylz2 (myosin light polypeptide 2)-mCherry double transgenic zebrafish line, with myf5 marking early myogenic commitment and mylz2 marking terminal differentiation (Xu et al., 2013a). Screening a library of 2400 small molecules identified 28 chemicals that perturbed muscle development and six that promoted myogenesis including three GSK3 inhibitors, two calpain inhibitors, and one adenylyl cyclase activator, forskolin. Combining the GSK3β inhibitor BIO, forskolin, and FGF2 induced efficient skeletal muscle specification of hiPSCs, indicating an effect conserved across species. Significantly, the highest myogenic differentiation efficiency was observed when the three factors were combined, compared to combinations of two factors only, suggesting a synergistic effect. The hiPSC-derived cells expressed muscle markers like *PAX7, MYF5, MYOD, MYOGENIN*, and *MYHC* and formed mature myotubes *in vitro*. Upon injection into cardiotoxin-injured TA muscles of immunocompromised mice, the hiPSC-derived myogenic progenitors contributed to muscle fibers and the SC pool in the host muscle.

## Summary

Major advances were made toward improving the generation and selection of SMCs and myogenic progenitors from hPSCs (Table 1). It is now possible to generate hPSC-derived myogenic progenitors in high purity and when using ectopic expression of myogenic factors to dramatically accelerate differentiation and improve efficiency. The amelioration of the DMD phenotype through the transplantation of hPSC-derived myogenic progenitors expressing exogenous PAX7 provides a proof of concept demonstrating the potential use of hPSCs for cell therapy targeting degenerative muscle diseases (Darabi et al., 2012). The use of small molecules is a promising alternative approach to drive myogenic differentiation of hPSCs into functional muscle progenitors without the use of transgenic interventions. In addition, small molecules facilitate single cell manipulation and increased clonogenicity of hiPSCs, and with recent advances in gene editing technologies represent important tools to facilitate gene correction and development of autologous cell replacement therapies for DMD and other muscular dystrophies (Valamehr et al., 2014; Li et al., 2015).

**Table 1:** **Methods for the myogenic differentiation of hPSCs**.

**Differentiation methods**		**Efficiency**	**Time**	**Myogenic stage**	**Scale up**	Complexity^**^
Transgenic	MyoD	High	Fast	Mostly mature	High^*^	High
	Pax7	High	Medium	Mostly progenitors	High	High
Non-transgenic	Co-culture	Low	Slow	Mixed	Medium	Medium
	EBs	Low	Slow	Mixed	Medium	Medium
	Small molecules	Medium	Medium	Mixed	Medium	Low (as monolayer)

* *When scale up is carried during the stage of mesodermal differentiation*.

** *Includes regulatory complexity*.

While hPSC-derived SMCs showed high engraftment and regenerative potential *in vivo*, their contribution remains localized as engrafted cells do not migrate more than few microns from the site of injection (Goudenege et al., 2012; Skuk et al., 2014). The limited migration of engrafted hPSC-derived SMCs/progenitors (and their primary counterparts) is a major limitation to their application as a cell therapy for systemic diseases like DMD, and other cell types that can be administered systemically are being investigated in preclinical and clinical studies (Tedesco et al., 2010; Meregalli et al., 2014). Besides SCs/ myoblasts, other muscle-associated cell types such as mesoangioblasts, pericytes, muscle side-population cells and CD133 + cells have been considered (Meregalli et al., 2014) and derived from hPSCs (Tedesco et al., 2012) as a potential treatment for muscle wasting. These cell types have variable features (e.g., systemic migration, proliferative potential, surface markers) and variable capacities to engraft and contribute to muscle repair. Therefore, one of the major challenges facing the field is identifying the optimal cell type(s) that is most suited to treat the multiple and variable diseases of the skeletal muscle system.

### Conflict of interest statement

The authors declare that the research was conducted in the absence of any commercial or financial relationships that could be construed as a potential conflict of interest.
